# Comparing the performances of SSR and SNP markers for population analysis in *Theobroma cacao* L., as alternative approach to validate a new ddRADseq protocol for cacao genotyping

**DOI:** 10.1371/journal.pone.0304753

**Published:** 2024-05-31

**Authors:** Angel Rafael Ramirez-Ramirez, Igor Bidot-Martínez, Khaled Mirzaei, Onisoa Léa Rasoamanalina Rivo, Miguel Menéndez-Grenot, Pablo Clapé-Borges, Georgina Espinosa-Lopez, Pierre Bertin

**Affiliations:** 1 Faculty of Agroforestry, University of Guantánamo, Guantánamo, Cuba; 2 Earth and Life Institute, Université catholique de Louvain, Louvain-la-neuve, Belgium; 3 Instituto de Investigaciones Agroforestales, Unidad de Ciencia y Técnica de Base—Baracoa, Baracoa, Guantánamo, Cuba; 4 Faculty of Biology, Department of Biochemistry, University of Havana, La Habana, Cuba; Arish university, Faculty of agricultural and environmental sciences, EGYPT

## Abstract

Proper cacao (*Theobroma cacao* L.) plant genotyping is mandatory for the conservation and use of the species genetic resources. A set of 15 international standard SSR markers was assumed as universal cacao genotyping system. Recently, different SNPs and SNP genotyping techniques have been exploited in cacao. However, a consensus on which to use has not been reached yet, driving the search for new approaches. To validate a new ddRADseq protocol for cacao genotyping, we compared the performances for population analysis of a dataset with 7,880 SNPs obtained from ddRADseq and the genotypic data from the aforementioned SSR set, using 158 cacao plants from productive farms and gene bank. Four genetic groups were identified with STRUCTURE and ADMIXTURE softwares using SSR and SNP data, respectively. Similarities of cacao ancestries among these groups allowed the identification of analogous pairs of groups of individuals, referred to as: G1_SSR_/G1_SNP_, G2_SSR_/G2_SNP_, G3_SSR_/G3_SNP_, G4_SSR_/G4_SNP_, whether SSRs or SNPs were used. Both marker systems identified Amelonado and Criollo as the most abundant cacao ancestries among all samples. Genetic distance matrices from both data types were significantly similar to each other according to Mantel test (p < 0.0001). PCoA and UPGMA clustering mostly confirmed the identified genetic groups. AMOVA and F_ST_ pairwise comparison revealed a moderate to very large genetic differentiation among identified groups from SSR and SNP data. Genetic diversity parameters from SSR (H_obs_ = 0.616, H_exp_ = 0.524 and PIC = 0.544) were higher than that from SNP data (0.288, 0.264, 0.230). In both cases, genetic groups carrying the highest Amelonado proportion (G1_SSR_ and G1_SNP_) had the lowest genetic diversity parameters among the identified groups. The high congruence among population analysis results using both systems validated the ddRADseq protocol employed for cacao SNP genotyping. These results could provide new ways for developing a universal SNP-based genotyping system very much needed for cacao genetic studies.

## Introduction

Cacao (*Theobroma cacao*) is a tropical tree species whose fermented seeds are used for cocoa powder, cocoa liqueur and cocoa butter production, that are very important products for chocolate, food and cosmetic industries. Cacao is often a small-scale farming crop and the livelihoods of 5–6 million farmers from Africa, Asia and Latin America depend on it, while another 40–50 million people are occupied by downstream bean processing industries worldwide [1].

Global cacao bean production has been estimated to 5,780,849 million tons and Africa countries are responsible for most of the world production with Ivory Coast, Ghana and Nigeria being the biggest producers in the region. In the past decade a ca. 25% increase was registered in both producing area and global bean production while less changes have been observed in global yield [2]. Most of the cacao produced in the world has strong acidic, astringent, intense cocoa flavor and is known as low quality or “bulk” cocoa, while only 5% of the total production has unique aroma and flavor characteristics classifying as fine cocoa [3].

Cacao genetic diversity in natural population is hosted in South and Central America forests and Upper amazon region, in the borders of Perú, Ecuador, Colombia and Brazil, is recognized as the center of origin of the species. The first attempts of cacao classification have been based on plant morphology identifying three groups: Forastero, Criollo and Trinitario which keep some connections with their geographical origin. Forastero group, found in South America, are robust, resistant productive plants whose beans are characterized by their astringency and bitterness. Criollo plants found in Central America are less productive, disease-susceptible but produce a high-quality fine aroma cocoa. Trinitario, firstly described in Trinidad and Tobago, resulted from the crossing between Forastero and Criollo plants and is characterized as productive plants of a good quality cocoa [4,5].

A new classification was introduced in 2008 by Motomayor et al. [6] based on Simple Sequence Repeat (SSR) DNA markers and recognized 10 genetic groups. This system confirmed Criollo group while Forastero plants were basically split into 9 groups: Amelonado, Contamana, Curaray, Guiana, Iquitos, Marañon, Nacional, Nanay and Purús. Trinitario plants are now recognized as hybrids between Amelonado and Criollo genetic groups though they may contain other Upper Amazon ancestries as well [7].

Other studies have also exploited SSR markers for various applications in cacao including: off-type detection, genetic diversity assessment and parentage analysis [8–10]. A set of 15 SSR markers was assumed as international standard microsatellites for cacao DNA fingerprinting [11,12] and it has been widely used for population analysis and cacao clone genetic classification, although carrying some disadvantages associated with SSR genotyping such as: allele size estimation, reproducibility and cost [13].

More recently, single nucleotide polymorphism (SNP) markers have been used for genetic studies in cacao and several SNP panels have been reported for different applications such as: population structure analysis, clone genetic classification, domestication history, association studies, among others [14–20]. Different genotyping techniques have been exploited to generate these SNP panels which include: MALDI-TOFF mass spectrometry [14], Illumina Infinity SNP Array [15], Whole Genome Sequencing [16], Fluidigram EP1^TM^ system [17], DArTseq [18], double-mismatch allele-specific (DMAS) qPCR [19] and Genotyping by Sequencing (GBS) based on double digestion [20]. However, most of these panels have not been systematically evaluated for optimum genotyping efficiency, as well as for population and sub-population classification and in some cases, proper separation of reference plants of cacao ancestry genetic groups has not been achieved. Therefore, the need remains for the identification of a SNP panel and genotyping method useful in the genetic classification of cacao clones [21,22]. This SNP panel would be later assumed as a universal SNP genotyping system similar to the international standard SSRs aforementioned. Counting with such system would provide a platform to strengthen the conservation and use of cacao genetic resources as well as the breeding and improvement programs worldwide leading to the identification of cacao clones with better agronomic and cocoa quality profile demanded by cacao farmers and chocolate makers [1,21,22].

We have applied ddRADseq technology (double digestion Restriction Assisted DNA sequencing), a reduced library representation genomic DNA sequencing technique [23], to assess the population structure and genetic diversity of cacao resources in Baracoa, eastern Cuba, which is the main cacao producing region of the country [24]. Few GBS protocols have been used in cacao genetic studies and even less exploited genomic DNA digestion with enzymes [18,20,25] as in ddRADseq [23]. Lachenaud et al. [18] used PstI and MseI enzymes to degrade cacao genomic DNA as part of a DArT sequencing approach; Osorio-Guarín et al. [20] digested genomic DNA with BsaXI and CspCI enzymes followed by a DNA fragment selection between 200–300 bp and a pair-end with 100 bp read length sequence strategy and finally, Adenet et al. [25] also employed PstI and MseI to digest DNA as Lachenaud et al. [18], while size selection (150–300 bp) and sequence strategy (single end, 150 bp) were different to other studies.

The applied ddRADseq protocol [24], differed from the abovementioned GBS protocols used in cacao [18,20,25], in respect to the enzyme combination used for genomic DNA digestion (EcoRI and NlaIII), the size of the selected DNA fragments (300–500 bp) and the sequencing strategy employed (pair-end, 150 bp) [24]. As with all GBS technologies, we identified thousands of SNP markers at a relatively low-cost surveying less than 10% of the cacao genome. However, some disadvantages are recognized for ddRADseq technology such as the need for high quality DNA preparations, allele dropout, PCR duplicates and variance in coverage, which could lead to genotyping errors. That is why it has been recommended to validate the variant detected with other methods, such as Sanger sequencing and real time-PCR, when high-impact applications are expected for the identified SNPs [26–28].

The high number of SNPs usually obtained in GBS experiments makes variant validation with the mentioned methods costly and time consuming. Alternatively, to validate the ddRADseq protocol used for cacao SNP genotyping, we compared the performances for population analysis of SNPs identified with this protocol versus the abovementioned 15 international standard SSR markers using 158 cacao plants from productive farms and gene bank. The results obtained from this research could provide new approaches for the development of a universal reliable SNP-based genotyping system for cacao genetic studies in the GBS era.

## Material and methods

### Plant material

Mature leaves were collected from 120 cacao plants from representative farms of the Baracoa region and 38 accessions from the National cacao gene bank preserved in Unidad de Ciencia y Técnica de Base-Baracoa / Instituto de Investigaciones Agroforestales (UCTB-Baracoa/INAF), making a total of 158 plants. All types of cacao plants found in Cuban cacao farms according to Bidot et al. [29], in terms of origin and reproduction mode were represented in the sampled plants, *i*.*e*.: grafted plants from UF clones, hybrids plants, TSH progeny and Cuban traditional cacao. A survey was applied to assess farms diversity in terms of: yield, soil properties, field condition, slope orientation, plant canopy diversity and, more importantly, cacao plant origin (grafted, hybrid, traditional). Seven farms were selected as representative among the surveyed ones. Twenty-nine plots containing 25 plants each were identified in the representative farms to cover all farm diversity detected. Five randomly selected plants from each plot were collected for analysis (S1 Table).

The 38 accessions from the cacao National gene bank included 33 recently prospected plants from Baracoa region, classified as Cuban traditional cacao [29], and 5 international recognized clones: UF29, UF613, UF60, UF677 and SCA6. Additionally, leaves of 35 cacao plants used as references of the 10 cacao ancestry genetic groups according to Motamayor et al. [6], were obtained from different sources for SSR genotyping (S2 Table).

Sequence data of 65 plants with high membership to the 10 cacao ancestry genetic groups [6], were downloaded and used as reference plants for SNP-based analyses. Using these data, Cornejo et al. [16] had already classified these plants as: Amelonado (10), Contamana (7), Criollo (4), Curaray (5), Guiana (7), Iquitos (6), Marañon (10), Nacional (4), Nanay (8) and Purús (4) (S3 Table). Only 3 of these plants were also references for SSR genotyping.

### SSR genotyping

DNA was extracted from 25 mg of dry leaves of the same 120 plants from Cuban farms and 38 ones from the Cuban collection, as well as of the 35 cacao genetic group reference plants–leading to a total of 193 plants–using DNeasy Plant Pro Kit and Tissue Lyser (QIAGEN, Germany) according to manufacturer recommendations. DNA concentration was estimated with a ND-1000 spectrophotometer (NanoDrop Technologies, USA).

SSR genotyping was performed using 15 microsatellite markers recommended as international standards for cacao genetic characterization because of their high levels of polymorphism, reproducibility and distribution throughout the genome [11,12]. Forward primers were 5’- labeled with the fluorescent dyes 6-carboxyfluorescein (6-FAM), 4,7,2’,4’,5’,7’-hexachloro-6-carboxyfluorescein (HEX) and 7’,8’-benzo 5’-fluoro-2’,4,7 trichloro-3-carboxyflourescin (NED).

SSR amplifications were performed with Type-it Microsatellite PCR kit according to producer recommendations (QIAGEN, Germany) in a Vapo.Protect Mastercycler^®^ thermal cycler (Eppendorf, Germany). PCR multiplex reactions were carried out in 10-μl total volume using primer pairs of three different SSR markers of different size ranges and dye labels for each multiplex reaction. Thermal cycling conditions consisted of initial denaturation at 95°C for 5 min, followed by 38 cycles of denaturation at 95°C for 30 s, annealing at 54°C for 45 s, and extension at 72°C for 2 min, with a final extension at 72°C for 5 min. Amplification products were detected using an ABI PRISM 3100 genetic analyzer (Applied Biosystems, USA) and a DS-32 (dye set F) matrix standard kit. Product sizes were scaled using a GeneScan 500 Rox standard (Applied Biosystems, USA). Amplified fragment sizes and intensities were visualized using the free software program Peak Scanner v1.0 (Applied Biosystems, USA).

### SNP genotyping

#### DNA purification, ddRADseq library preparation and sequencing

DNA purification and ddRADseq library preparation were performed as described [24]. DNA was purified using a CTAB based protocol described for plant leaves with high polysaccharides and polyphenols content [30]. Shortly, dry cacao leaves (25 mg) were converted into fine powder. The powder was washed twice with cold sorbitol buffer (0.35 M Sorbitol, 100 mM Tris-HCl, 5 mM EDTA, 1% PVP-40, 1% 2-mercaptoethanol, pH = 8.0). Followed by DNA extraction with pre-warmed (65°C) extraction buffer (3% CTAB, 100 mM Tris-HCl, 20 mM EDTA, 3 M NaCl, pH 8.0) and 20 μL Proteinase K (10 mg/mL), 35 μL of Sarkosyl 30% and 30 mg of PVPP were added to each tube. After 1 h incubation at 65°C, 800 μL of Chloroform:isoamyl alcohol (24:1) were added and mixed by inversion for 15 minutes. Aqueous phase was collected and transferred to a fresh clean tube, where volumes equivalent to 1/10 times of 3 M NaAc pH 5.2 and 2/3 times of cold isopropanol (-20°C) were added to the homogenates. DNA pellet was collected by centrifugation and washed with 70% ethanol. Supernatants were discarded and tubes were left open to dry at room temperature. Pellets were dissolved in 100 μl of TE buffer containing DNase-free RNase A (200 μg/mL) by incubation at 37°C until complete dissolution. A DNA cleaning step was implemented using silica columns of the DNeasy Plant Pro Kit from QIAGEN (Germany). Final DNA preparation was eluted with 65 μL of TE buffer and kept at -20°C until use. DNA integrity was confirmed by agarose gel electrophoresis at 1.5%. DNA was quantified with Qubit^TM^ dsDNA BR Assay Kit (Thermo Fisher Scientific, USA) according to the manufacturer´s instructions.

Sequencing libraries were prepared as described [23]. DNA were digested with EcoRI HF and NlaIII using the Cut Smart Buffer (NEB, USA) at 37°C overnight. Digested DNA was put to ligation for 8 h at 16°C with adaptors designed for ddRADseq sequencing libraries [23] using T4 ligase. After ligation, samples were conveniently pooled to conform sub-libraries and purified with magnetic beads (Promega, USA). DNA fragments between 300–500 bp were selected using a BluePippin instrument (Sage Science Inc., USA). Size-selected DNA preparations were Polymerase Chain Reaction (PCR)-enriched using Phusion® High-Fidelity DNA Polymerase (NEB, USA). PCR products were magnetic bead-purified and combined to get ddRADseq sequencing libraries. Libraries were sequenced on a HiSeq2500 instrument (Illumina, USA) following a pair-end strategy with a read length of 150 bp.

#### Data processing and SNP calling

FastQC v0.11.9 was used to check DNA sequence quality. Demultiplexing was performed with process_radtags from Stacks v2.5 and the reads with an average base quality (Q) score lower than 25 in a single 15 nt window were discarded [31,32]. Nucleotides from the 5’ (8) and 3’ (15) ends were removed with TrimGalore!/cutadapt [33], along with a 3’ quality trimming to remove bases with Q < 25. Only paired reads longer than 75 nt were kept. Sequences were aligned to Matina 1–6 cacao reference genome [34] using bwa mem from BWA v0.7.17 [35]. *sam* files were converted into *bam* files and later cleaned, fixed, sorted and RG group added by combining SAMtools v1.10 and Picard tools v2.18.25 [36,37].

The bam files of the 158 plants from Cuban farms and National gene bank were used for SNP calling by combining the tools: *BaseRecalibrator*, *HaplotypeCaller*, *CombineGVCFs* and *GenotypeGVCFs* from *GATK* v4.2.0.0 [38]. Raw SNPs were hard-filtered with the *VariantFiltration* tool following GATK hard filtering recommendations [39]. An additional filtering step was added to select SNPs with high representation among the samples by using VCFtools v0.1.16 and the filtering options: maf > 0.05, site missing < 5%, biallelic, SNP depth coverage: 10–80, SNP spacing > = 1,000 nt [40].

Final SNP dataset was obtained by intersecting filtered SNP dataset and a SNP dataset built from the sequence data of the 65 cacao reference plants representing the 10 cacao ancestry genetic groups (Reference SNP dataset; S3 Table) [6,16]. This intersection aimed to look for coincidences between these two datasets, keeping only the coincident SNPs. The final SNP dataset (henceforth SNP dataset or SNPs) contained 7,880 variants and was employed for population analysis studies. SNP dataset Transition/Transversion ratio, missing data and depth of coverage on individual based were estimated with VCFtools. The SNP distribution along the cacao chromosomes was analyzed with the function CMplot (R package “CMplot” v4.5.0) using a 1 Mb window size [41,42].

### Population analysis

#### Population structure

Model based clustering was performed for both types of data although using different softwares: STRUCTURE v2.3.4 for SSR [43] and ADMIXTURE v1.3 for SNPs [44]. The high computing demand of STRUCTURE for analyzing large SNP panels made us discard it for SNP data, whereas ADMIXTURE only works for SNP datasets especially large ones. For SSR data, genetic groups were identified with model-based Bayesian clustering method as implemented in STRUCTURE v2.3.4 with the aid of parallel_structure function from ParallelStructure package v1.0 form R statistical language version 4.2 [42,45]. Ten independent runs from K = 1 to K = 12 with 200,000 iterations with an initial burn-in period of 100,000 iterations were performed. The most probable K was determined by the graphical method of Evanno et al. [46]. For cacao ancestry estimation according to Motamayor et al. [6], the prior population information option of STRUCTURE was used (“usepopinfo”) with the SSR genotyping data of the 35 cacao ancestry genetic group reference plants (S2 Table). The proper separation of these 35 reference plants into the expected cacao ancestry genetic group was assessed before ancestry estimation based on SSR data (S4 and S5 Tables, S1–S3 Figs).

In the case of SNP data, genetic groups were detected with the maximum likelihood method implemented in ADMIXTURE v1.3 software by a 5-fold cross-validation procedure under penalized (-l 500, -e 0.2) and random seedling (-s time) conditions [16,44,47]. Twenty replicas for K values ranging from 1 to 12 were performed following Liu et al. recommendations [48]. Best K value was determined by the minimum cross-validation error criteria suggested by software developers [47]. Membership to cacao ancestry genetic groups of Motamayor *e*t al. [6] of each individual was calculated by running ADMIXTURE under supervised mode with the aforementioned penalized options. A version of the Reference SNP dataset with the 65 cacao references containing the same SNPs positions as the final SNP dataset was used for training purposes (S3 Table). The capacity of the 7,880 SNPs from the final SNP dataset to properly separate reference plants into the expected cacao ancestry genetic group was confirmed before ancestry analysis (S4 and S5 Tables, S1–S3 Figs).

Five recognized cacao clones were used to compare cacao ancestry detection at the individual level: SCA6, UF29, UF613, UF650 and UF677. Cacao ancestries of these clones from the International Cocoa Germplasm Database (ICGD) [49] and published data [17] were also collected for comparison purposes.

#### PCoA and UPGMA clustering

Principal coordinate analysis (PCoA) and clustering based on UPGMA were applied to confirm the genetic groups identified with the model-based approaches among the 158 plants from Cuban farms and national gene bank. To that purpose genetic distance matrices were built from SSR and SNP data based on the percentage of allelic differences with diss.dist function from poppr package v2.9.3 in R [50,51]. Mantel test for matrix similarity with 10,000 permutation and Spearman correlation analysis were executed for matrices comparison with mantel.test function from ape package v5.6–2 and cor.test from stats package v4.1.2, respectively [42,52]. PCoA was performed from these distance matrices with pcoa function from ape package and using the cailliez correction for negative values. UPGMA clustering was performed from the genetic distance matrices and trees were built using a bootstrapping procedure with 1000 replicas with aboot function of poppr package v2.9.4 and visualize with ggtree package v3.10.0 from R statistical language version 4.2 [42,53].

### Differentiation between groups and genetic diversity

AMOVA test was used to assess genetic variation among the genetic groups identified by model-based softwares as source of variability, using the 158 plants from Cuban farms and Cuban gene bank. Analysis was done according to Excoffier et al. [54] with the function poppr.amova from poppr package. Test significance was estimated by randtest function of ade4 package v1.7–19 with 999 permutations [55,56]. F_ST_ pairwise comparison for identified genetic groups were calculated by Weir & Cockerham procedure [57] with pairwise.WCfst function of hierfstat package v0.5–11 [58], 10,000 bootstraps were performed for confidence intervals (95%) and p-value estimation with boot.ppfst function from the same package.

Genetic diversity parameters, *i*.*e*. observed (H_obs_) and expected (H_exp_) heterozygosities, and polymorphic information content (PIC) were estimated with adegenet v2.1.7, polysat v1.7–7 and poppr v2.9.4 packages from R program [50,51,59–61]. Effective number of allele (*Ne*) was calculated from allele frequencies (*pi*) by:

$$Ne=1/\Sigma {pi}^{2}$$

## Results

### SSR and SNP genotyping

All SSR alleles from the 158 samples under study were successfully recorded except for two plants which failed to amplify mTcCIR11 locus, representing around 0.17% of missing data. In total, 109 alleles were identified among the 15 microsatellites. mTcCIR7 had the lowest number of alleles—4—while mTcCIR6 and mTcCIR33 were the most polymorphic ones with 11 different alleles each. The 35 plants representing the reference plants of cacao genetic groups by Motamayor et al. [6] were also analyzed with this marker set, showing a low—but slightly higher—percent of missing data (0.57%) and a higher number of alleles than the samples (127) (S6 Table).

Total raw SNPs accounted for 988,300 which were reduced first to 9,252 after filtering and later to 7,880 because of the intersection with the SNP dataset containing the 65 references of cacao ancestry genetic groups according to Motamayor et al. [6]. Missing data and depth of coverage averages of the final SNP dataset (7,880 SNPs) were 1.65% and 20.3X, respectively. Hard-filtering per SNP variables showed the expected distribution (S4 Fig) [39] and the transition/transversion ratio of the final SNP dataset (1.591) were similar to the 1.682 value reported for another cacao SNP panel (S7 Table) [62]. These results strongly supported the high quality of the SNP dataset obtained with the ddRADseq protocol. SNPs were distributed along the 10 cacao chromosomes with an average of 23.8 SNPs per Mb. Chromosome 1 had the highest SNPs density (26.8) and chromosome 7 showed the lower count of SNPs per Mb (20.8) (S4 Fig).

Only a low percent of the final SNPs (83 out 7,880) matched with the SNPs contained in a developed 15K SNP array for cacao [63] which has been used as reference for whole genome sequencing experiments [16] and for selecting SNP markers reflecting population origin for cacao identification [22]. Also, low number of SNPs (3) were identified as coincident when the final SNPs were compared with a 96 SNP set that has been widely used for offtyping in cacao breeding programs [64]. However, the characterization of SSR and SNP data (S6–S8 Tables, S4 and S5 Figs), particularly the low missing data values obtained, revealed both datasets were suitable for population analysis studies and provided a proper platform for genotyping system comparison and hence ddRADseq protocol validation.

### Genetic group identification based on SSR and SNP data

**Fig 1:**
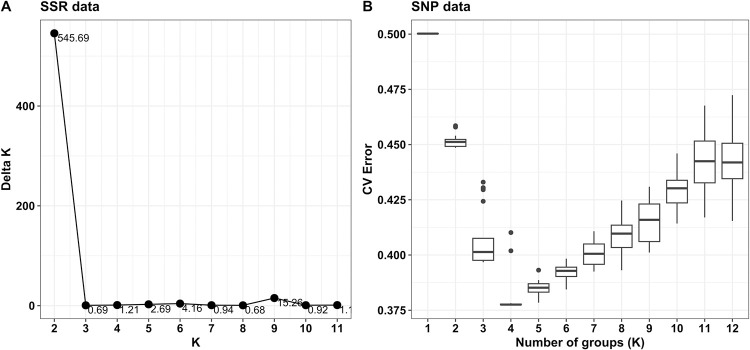
Estimation of best K from both SSR and SNP data. (A) Delta K (ΔK) values for SSR data according to Evanno method [46]. (B) CV error at the different K values for SNP data following Alexander et al. [44,47]; horizontal lines represent the median, boxes stand for the 25 and 75% percentiles, vertical lines point to minimum and maximum values and the dots are “outlier” data.

**Fig 2:**
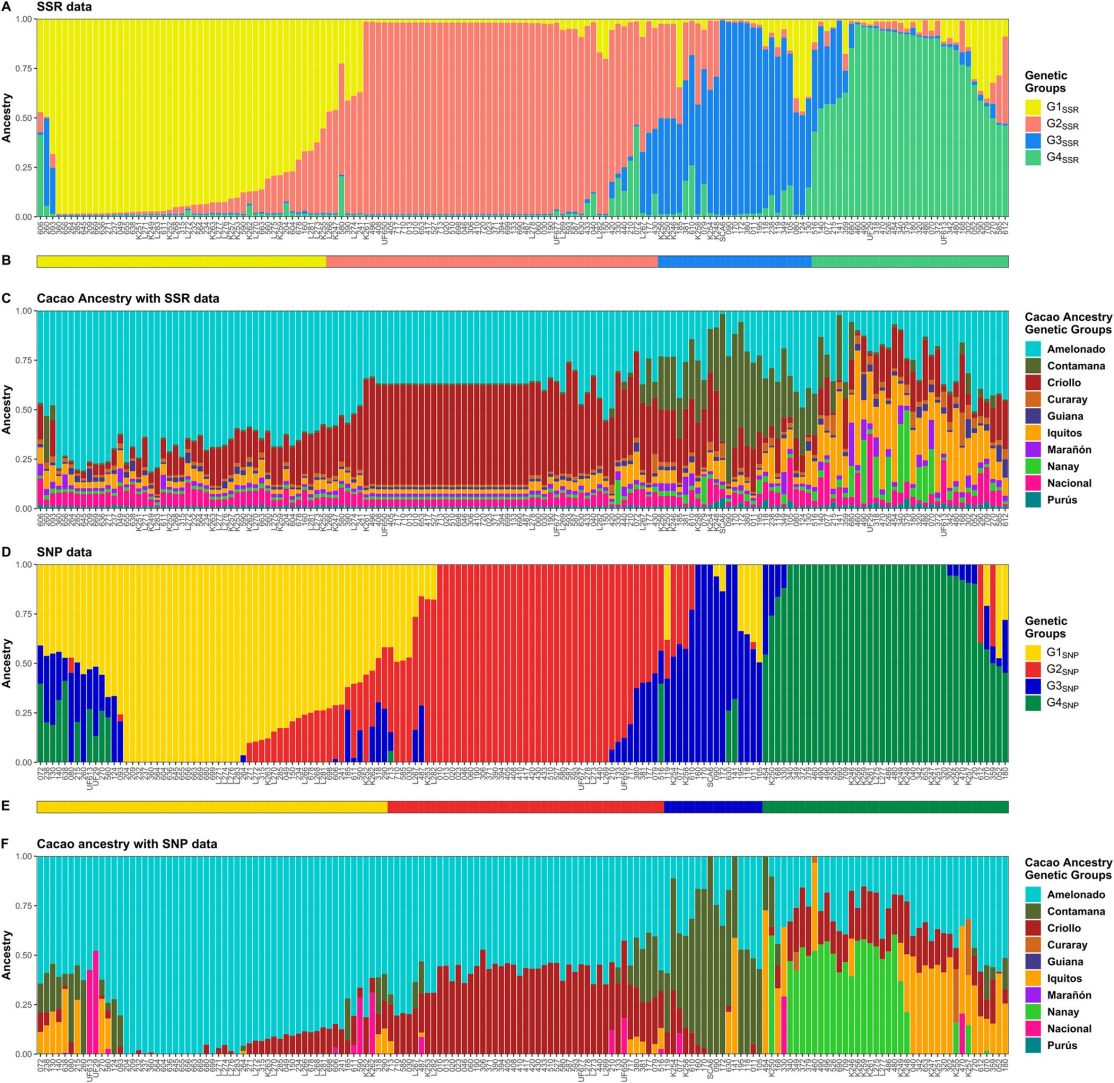
Memberships based on SSR or SNP data and assuming four groups. Each column represents an individual. (A) Membership according to STRUCTURE (SSR data) to specific genetic groups (G1_SSR_, G2_SSR_, G3_SSR_ and G4_SSR_). (B) Assignment to groups based on SSR data: G1_SSR_ (light yellow-green), G2_SSR_ (light red), G3_SSR_ (light blue), G4_SSR_ (light green). (C) Membership to cacao ancestry genetic groups identified by Motamayor et al. [6] using SSR data and STRUCTURE with “usepopinfo” option. (D) Membership according to ADMIXTURE (SNP data) ran under cross validation mode to specific genetic groups (G1_SNP_, G2_SNP_, G3_SNP_ and G4_SNP_). (E) Assignment to groups based on SNP data: G1_SNP_ (dark yellow), G2_SNP_ (dark red), G3_SNP_ (dark blue), G4_SNP_ (dark green). (F) Membership to cacao ancestry genetic groups identified by Motamayor et al. [6] using SNP data and ADMIXTURE under supervised mode. Plots were generated using ggplot2 and ggpubr packages from R program.

Best K value estimation based on recognized procedures differed from both types of data. K = 2 revealed as the most probable for SSR data according to Evanno method [46] and K = 4 for SNP data according to CV error minimum [47] (Fig 1). However, a high similarity was observed when plotting the membership matrices obtained from assuming K = 4 for both SSR and SNP data (Fig 2A and 2D). Likewise, membership matrices to cacao ancestry genetic groups according to Motamayor et al. [6] were highly similar with both SSR and SNP data (Fig 2C and 2F). This led to assume four as the most probable number of genetic groups in both marker types. For group assignment, samples were allocated to the group showing the highest membership value, originating four SSR groups (G1_SSR_, G2_SSR_, G3_SSR_, G4_SSR_). In the same way, four SNP groups were defined: G1_SNP_, G2_SNP_, G3_SNP_ and G4_SNP_ (Fig 2B and 2E).

Pairs of analogous groups were identified when comparing membership plots (Fig 2): G1_SSR_ / G1_SNP_, G2_SSR_ / G2_SNP_, G3_SSR_ / G3_SNP_ and G4_SSR_ / G4_SNP_. Significant and positive correlations (Spearman correlation, p < 0.0001) were detected between membership coefficients to SSR and SNP analogous groups: G1_SSR_-G1_SNP_ (rho = 0.61), G2_SSR_-G2_SNP_ (0.59), G3_SSR_-G3_SNP_ (0.61) and G4_SSR_-G4_SNP_ (0.40). The correspondence among analogous groups was also assessed by computing the coincidence (Table 1), which varied from 55.56 for G4_SSR_-G4_SNP_ to 76.77% for G2_SSR_-G2_SNP_. These percentages improved up to 82.76%, when neighbor groups (Fig 2 and Table 1) were combined for computing (G1_SSR_/G2_SSR_ vs G1_SNP_/G2_SNP_ and G3_SSR_/G4_SSR_ vs G3_SNP_/G4_SNP_).

**Table 1:** Number of individuals, coincident count and coincidence (%) between analogous groups identified from SSR and SNP data.

Data type	G1	G2	G3	G4	G1 & G2	G3 & G4
SSR	47	54	25	32	101	57
SNP	57	45	16	40	102	56
Coincident	37	38	12	20	84	39
Coincidence (%)	71.15	76.77	58.54	55.56	82.76	69.03

**SSR** and **SNP** refer to the data type. **G1** refers to analogous group 1 from SSR (G1_SSR_) and SNP (G1_SNP_), similar approach is applied for **G2**, **G3** and **G4**. **G1 & G2** refers to the combination of G1_SSR_/G2_SSR_ and G1_SNP_/G2_SNP_ similar is applied for **G3 & G4**. Percentage of coincidence for analogous groups were estimated against the average counting of the analogous groups under consideration.

Cacao ancestry analysis of all plants revealed a similar pattern of average memberships to ancestry genetic groups of Motamayor et al. [6] using both data types. In SSR, Amelonado (0.423) was the more abundant ancestry followed by Criollo (0.243), and to a lesser extent Iquitos (0.083), Contamana (0.075) and Nacional (0.060). Almost similarly, detected ancestries using SNPs were Amelonado (0.578), Criollo (0.169), and to a lesser extent Contamana (0.094), Iquitos (0.070) and Nanay (0.067). A high correspondence was identified between SSR and SNP analogous groups based on their cacao ancestry average (Table 2). G1_SSR_ (0.673) and G1_SNP_ (0.837) showed the highest kinship to Amelonado among their respective groups while G2_SSR_ and G2_SNP_ were mainly conformed by hybrids between Amelonado and Criollo, though the proportion of the contributing ancestries were different: G2_SSR_ hybrids had more ancestry from Criollo (0.442) than Amelonado (0.384) while in G2_SNP_ hybrids, Amelonado contribution (0.576) was greater than Criollo (0.365). G3_SSR_ and G3_SNP_ excelled by the presence of Contamana ancestry (G3_SSR_ average: 0.335 and G3_SNP_ average: 0.575) mostly as hybrids with Amelonado. G4_SSR_ and G4_SNP_ were highly mixed groups sharing ancestry from Amelonado, Criollo and Iquitos, although G4_SNP_ had also an important contribution from Nanay (0.268) which was less evident for G4_SSR_ (0.087).

**Table 2:** Average membership to cacao ancestry genetic groups according to Motamayor et al. [6] of the analogous genetic groups identified with STRUCTURE (SSR) or ADMIXTURE (SNPs) assuming K = 4.

Groups	Amelonado	Contamana	Criollo	Iquitos	Nanay	Nacional
G1_SSR_	0.673	0.014	0.118	0.045	0.015	0.064
G1_SNP_	0.837	0.049	0.051	0.033	0	0.029
G2_SSR_	0.384	0.017	0.442	0.036	0.016	0.046
G2_SNP_	0.576	0.04	0.365	0.01	0	0.009
G3_SSR_	0.249	0.335	0.173	0.066	0.029	0.062
G3_SNP_	0.309	0.575	0.06	0.05	0	0.007
G4_SSR_	0.261	0.06	0.145	0.232	0.087	0.079
G4_SNP_	0.32	0.027	0.159	0.197	0.268	0.012

Curaray, Guiana, Marañon and Púrus ancestries were calculated but not represented because memberships to them were lower than 5% for all the identified groups.

In general, the per group average of the more contributing ancestries estimated with SNP data were higher than with SSR data except for Criollo ancestry in G2 groups. Probably, because with SSR data it could be detected the presence of small contributions from all cacao ancestries, apart from the major contributing ones, in almost every sample; *e*.*g*. small contributions from Nacional and Iquitos ancestries were evident in all samples (Fig 2C). As result of these small contributions, the membership coefficients of the more contributing ancestries for every sample came to less. Such pattern was not detected in the SNP-based ancestry membership coefficients (Fig 2F). However, the fact that with both systems, analogous pairs of groups were identified based on their cacao genetic ancestry supported the use of both genotyping systems for cacao population structure analysis and hence contributed to validate the ddRADseq protocol exploited for SNP genotyping.

#### Ancestry of recognized clones using SSR and SNP data

Five plants included in this study are recognized cacao clones: UF29, UF613, UF650, UF677 and SCA6; some of them are currently used in Cuban cacao farming practices [65]. Cacao genetic group ancestries according to Motamayor et al. [6] were established for these clones (Table 3), using both SSR and SNP data, and compared to their ancestries determined by SNP genotyping from either published study [17] or deposited data in the International Cocoa Germplasm Database (ICGD) [49]. For UF29 and UF613, both SSR and SNP data were able to identify Amelonado and Nacional ancestries as the more contributing ones, similarly to other studies. No other ancestry was detected for these clones with SNP data, while Iquitos was also detected with SSR data, opposite to either published or deposited data (Table 3). Nacional ancestries in these clones were the highest found among all the samples using either SSR (0.356 (UF29), 0.218 (UF613)) or SNP (0.522, 0.426) data. Surprisingly, these two clones were located in G4_SSR_ using SSR data but were assigned to G1_SNP_ with SNP data. This result might be related to the detection of Iquitos ancestry with SSR data in these clones as already mentioned.

**Table 3:** Membership coefficients to cacao ancestry genetic groups by Motamayor et al. [6] of recognized cacao clones.

Source	Clone	Amelonado	Contamana	Criollo	Curaray	Iquitos	Nacional
**SSR**	UF29	0.203	0.012	0.013	0.042	0.262	0.356
**SNPs**	UF29	0.478	0.000	0.000	0.000	0.000	0.522
**ICGD**	UF29	0.524	0.002	0.002	0.002	0.004	0.444
**CATIE**	UF29	0.510	0.010	0.000	0.000	0.010	0.450
**SSR**	UF613	0.370	0.012	0.027	0.079	0.203	0.218
**SNPs**	UF613	0.574	0.000	0.000	0.000	0.000	0.426
**ICGD**	UF613	0.596	0.005	0.047	0.004	0.005	0.328
**CATIE**	UF613	0.570	0.010	0.040	0.000	0.010	0.350
**SSR**	UF650	0.368	0.008	0.503	0.011	0.022	0.036
**SNPs**	UF650	0.427	0.000	0.390	0.000	0.000	0.183
**CATIE**	UF650	0.550	0.000	0.430	0.000	0.000	0.000
**SSR**	UF677	0.355	0.009	0.468	0.018	0.047	0.045
**SNPs**	UF677	0.546	0.000	0.454	0.000	0.000	0.000
**ICGD**	UF677	0.502	0.002	0.475	0.004	0.003	0.002
**SSR**	SCA6	0.016	0.645	0.010	0.075	0.109	0.045
**SNPs**	SCA6	0.000	0.999	0.000	0.000	0.000	0.000
**ICGD**	SCA6	0.001	0.978	0.002	0.002	0.001	0.010

**Source** refers to the origin of the data, **SSR** and **SNPs** were obtained in this study, **ICGD**: Deposited on International Cacao Germplasm Database [49] and **CATIE** (Centro Agronómico Tropical de Investigación y Enseñanza): Published on Mata-Quiros et al. [17]). **Clone** is the identifier of the cacao clone under study. Guiana, Marañon, Nanay and Purús ancestries were not considered because memberships to them were lower than 5% for all the clones.

In the case of UF650 and UF677, both marker systems as well as reported data, identified them as hybrids of Amelonado and Criollo but the contributions were different according to the marker system used. With SSR data, hybrids contained more Criollo (Q_Ave_ = 0.485) ancestry than Amelonado (0.361) while it was the other way around with SNP markers (Q_Amelonado_ = 0.486, Q_Criollo_ = 0.422). Intriguingly a small portion of Nacional ancestry was detected in UF650 (0.183) with SNP data (Table 3). Finally, using SNP markers, SCA6 was identified as a very strong member of the Contamana cacao genetic group (Q = 0.999) similarly to ICGD deposited data, while using SSR data, it achieved the highest membership to this same cacao genetic group (0.645) among all SSR-genotyped samples.

Remarkably, the presence of small contributions of other ancestries, apart from the more important ones, with SSR data was also evident in these five cacao clones as with group average ancestries (Tables 2 and 3); which resulted in lower values of membership coefficients for the more contributing ancestries with this dataset in comparison with SNP data. These results support SNP as more suitable marker system than SSR for cacao ancestry estimation of individual plants which in turn could allow a more precise classification based on ancestry information of the plants under study.

### Genetic distances between pairs of samples

Genetic distance matrices based on allelic differences between pairs of plants were built from both SSR and SNP data. SSR-based distances varied from 0 to 0.833 with a mean of 0.442 while SNP-based distances ranged from 0.014 to 0.437 with 0.221 as average. Matrices comparison using Mantel’s permutation test for similarity detected a significant correlation between them (p < 0.0001) which was also confirmed by the positive and significant correlation between genetic distance estimates from SSR and SNP data revealed by Spearman correlation coefficient (rho = 0.475, p < 0.0001).

### PCoA and UPGMA based on genetic distances

**Fig 3:**
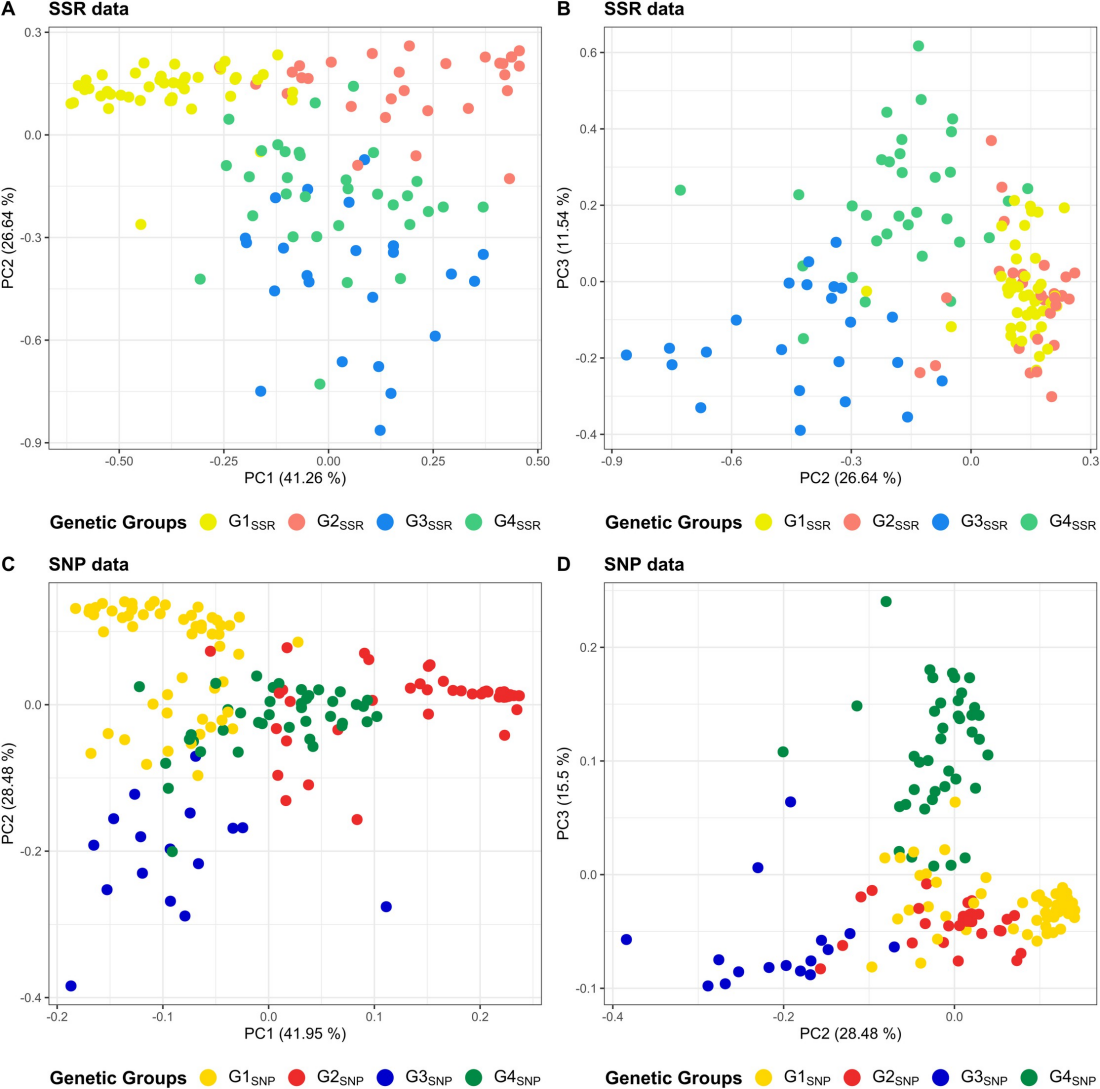
Principal coordinate analysis (PCoA) bi-plots of cacao plants based on SSR and SNP data. (A) PC1 and PC2, (B) PC2 and PC3 bi-plots using SSR data. (C) PC1 and PC2, (D) PC2 and PC3 bi-plots using SNP data. PCoA was performed from genetic distance matrix based on allelic differences using either SSR or SNP data. Coloring is based on group membership from STRUCTURE or ADMIXTURE programs assuming K = 4 (Fig 2). Plots were generated using ggplot2 and ggpubr packages from R program.

Principal coordinate analyses using abovementioned genetic distance matrices were executed to corroborate previously identified groups. The three principal components explained 79.44% and 85.93% of the variation for SSR and SNP data, respectively (Fig 3). Sample distributions on bi-plots were alike for both types of data and allowed the separation of the genetic groups identified by either STRUCTURE or ADMIXTURE software. Firstly, PC1-PC2 achieved a proper separation between G1_SSR_ and G2_SSR_ (Fig 3A), as well as G1_SNP_ and G2_SNP_ (Fig 3C), from each other and from the remaining groups, though a better grouping was observed with SSR data. Secondly, PC2 and PC3 clearly put apart the remaining groups identified with both SSR and SNP data: G3_SSR_ and G4_SSR_ (Fig 3B), G3_SNP_ and G4_SNP_ (Fig 3D).

**Fig 4:**
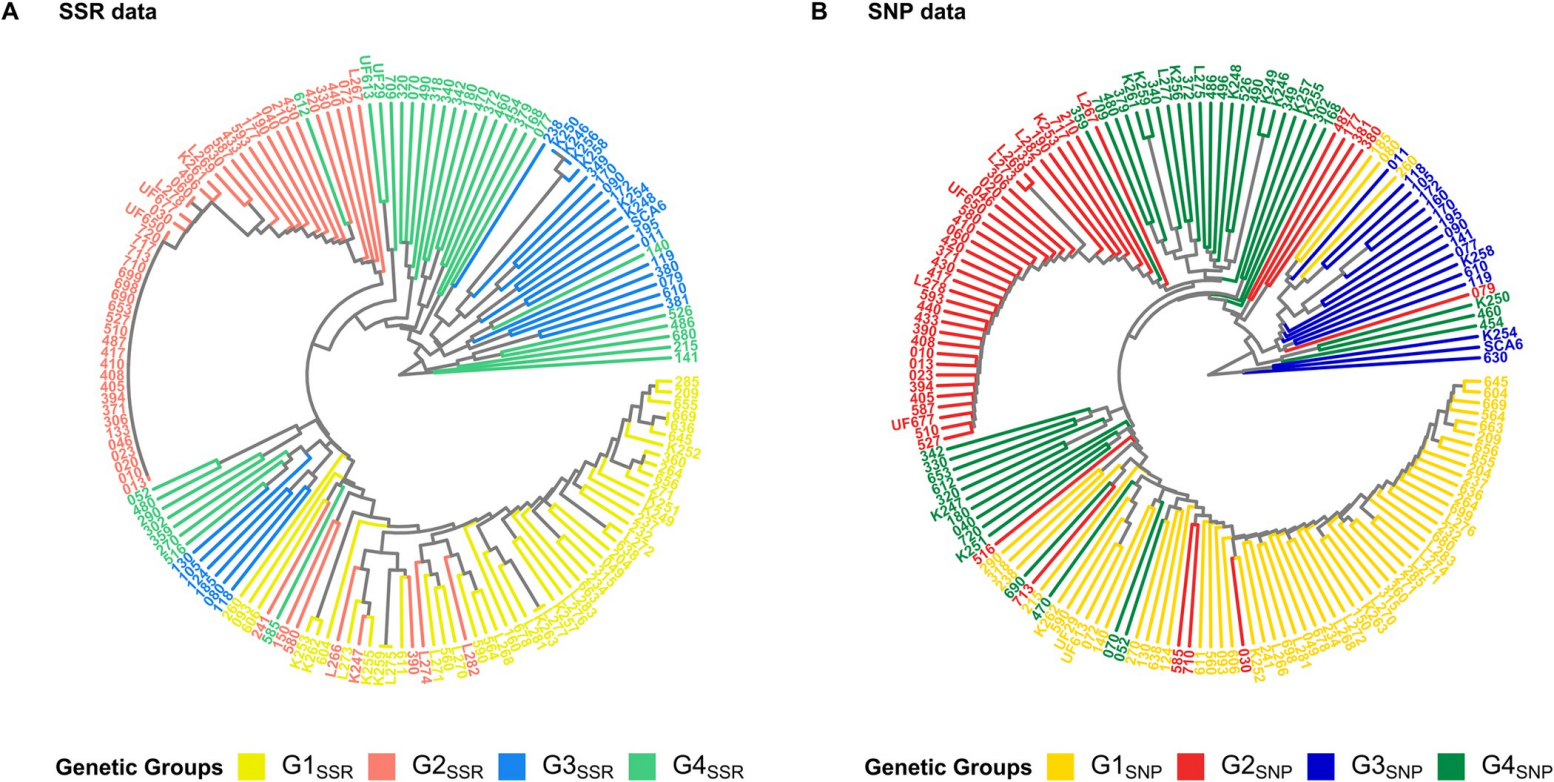
Dendrograms using SSR (A) or SNP (B) data of the 158 cacao plants. Clustering by UPGMA was built from genetic distance matrices based on allelic differences. Coloring is based on group membership from STRUCTURE or ADMIXTURE programs assuming K = 4. Plot was generated using ggtree and treeio packages from R program.

Clustering analysis by UPGMA from genetic distance matrices was also assessed to confirm groups conformation (Fig 4). Dendrograms revealed an adequate correspondence between the clustering from both data types and with the already identified groups with model-based approach and PCoA (Figs 2 and 3). Clustering worked mostly well for G1 and G2 groups for both marker systems, though some plants of these groups were allocated differently, especially in the groups from SNP data (G1_SNP_ and G2_SNP_) (Fig 4), similar to PCoA results. For G3, a better clustering was achieved for G3_SNP_ samples than for G3_SSR_ ones. The six G3_SSR_ samples (080, 105, 118, 124, 130 and 185) that failed to cluster with most G3_SSR_ samples shared a higher ancestry proportion from Amelonado (Average ~ 0.40) than the G3_SSR_ group average (0.249). In the case of G4_SSR_ and G4_SNP_ a less efficient grouping was detected since samples were split in at least two subgroups in the dendrograms, though a similar distribution pattern of the subgroups could be identified using both types of data (Fig 4). G4 subgroups laying between G1 and G2 branches in both dendrograms, had a lower contribution of the ancestries Iquitos and Nanay than the G4 average for SSR (Iquitos/Nanay subgroup average: 0.237 vs G4_SSR_ average: 0.319) as well as for SNP data (Iquitos/Nanay subgroup average: 0.344 vs G4_SNP_ average: 0.465). G4 groups excelled by the presence of Iquitos and Nanay ancestries among their members; therefore, changes in the contribution of these ancestries may impact the grouping of these plants. The high mix of plants contained in G4_SSR_ and G4_SNP_ groups is another reason behind their grouping pattern in the dendrograms.

The high congruence detected between clustering analysis either PCoA or UPGMA and the results from population structure analysis using SSR and SNP datasets confirmed both genotyping systems as valid for cacao genetic studies and particularly the ddRADseq protocol described for cacao SNP genotyping.

### Group differentiation and genetic diversity using SSR and SNP data

Variation among plants and genetic groups identified by either STRUCTURE or ADMIXTURE was assessed by AMOVA test (Table 4). For both types of data, the larger contribution to variation came from within groups, accounting for 66.01% (SSRs) and 72.41% (SNPs) of the total variation. Accordingly, between groups variation was slightly higher for SSR (33.99%) than for SNPs (27.59%). These last values were enough to support a good differentiation among groups either based on SSR or on SNP data, as attested by F_ST_ pairwise comparison results (Table 5). In both cases, all F_ST_ values were significant (p < 0.001) and the average F_ST_ from SSR and SNP data were quite similar to each other (0.166 vs 0.161 for SSRs and SNPs, respectively). Coincidentally, the highest F_ST_ values were detected between analogous groups G1_SSR_-G3_SSR_ (0.283) and G1_SNP_-G3_SNP_ (0.260), and four out of six F_ST_ values from both data types supported a large (0.15 < F_ST_ < 0.25) to very large (F_ST_ > 0.25) genetic differentiation. The remaining values attested a moderate differentiation between the groups (0.05 < F_ST_ < 0.15), *i*.*e*. G2_SSR_-G4_SSR_ (0.103), G3_SSR_-G4_SSR_ (0.072), G1_SNP_-G4_SNP_ (0.118) and G2_SNP_-G4_SNP_ (0.087) (Table 5).

**Table 4:** AMOVA results from SSR and SNP data assuming 4 groups.

	SSR data				SNP data			
Source of Variation	Df	SS	Sigma	Variation (%)	Df	SS	Sigma	Variation (%)
Between Groups	3	179	1.48	33.99	3	39,730.51	329.04	27.59
Within Groups	154	442	2.87	66.01	154	132,959.65	863.37	72.41
Total	157	620	4.34	100	157	172,690.16	1,192.42	100

**Df:** Degree of freedom, **SS:** Square Sum, **Sigma** represents the variance, (p < 0.001).

**Table 5:** F_ST_ pairwise comparison of the groups identified from SSR and SNP data.

SSR data				SNP data			
	G1_SSR_	G2_SSR_	G3_SSR_		G1_SNP_	G2_SNP_	G3_SNP_
**G2**_**SSR**_	0.211			**G2**_**SNP**_	0.160		
**G3**_**SSR**_	0.283	0.149		**G3**_**SNP**_	0.260	0.193	
**G4**_**SSR**_	0.176	0.103	0.072	**G4**_**SNP**_	0.118	0.087	0.149

All Fst values were significant (p < 0.001).

A moderate to low genetic diversity was detected among individuals and groups by the estimated number of alleles (N_a_), effective number of alleles (N_e_), observed and expected heterozygosity (H_obs_, H_exp_) and polymorphic information content (PIC) from either SSR or SNP data. Overall genetic diversity parameters obtained for SSR data (N_e_ = 2.331, H_obs_ = 0.616, H_exp_ = 0.524, PIC = 0.544) were higher than those for SNP data (1.427, 0.288, 0.264, 0.23) (Table 6). The larger number of alleles (Na) per locus found for SSR (7.267) in respect to SNP (2) is common when SSR and SNP markers are compared to each other and is probably causing the differences in genetic diversity parameters detected between both marker types [66–68]. Per groups genetic diversity parameters revealed some correspondences, G1_SSR_ and G1_SNP_ showed the lowest values for the assessed parameters among their respective genetic groups while group ordering based on H_obs_ decreasing value were the same for each type of data: G2_SSR_ (0.823)—G4_SSR_ (0.725)—G3_SSR_ (0.647)—G1_SSR_ (0.267) and G2_SNP_ (0.395)—G4_SNP_ (0.308)—G3_SNP_ (0.294)—G1_SNP_ (0.154). Ne, Hexp and PIC values for G2_SNP_, G3_SNP_ and G4_SNP_ groups had a lower variation between each other than the one detected between G2_SSR_, G3_SSR_ and G4_SSR_ groups (Table 6).

**Table 6:** Genetic diversity of all samples and identified genetic groups with SSR and SNPs data.

**SSR data**						
**Group**	**N**	**Na**	**Ne**	**Hobs**	**Hexp**	**PIC**
G1_SSR_	47	3.47	1.450	0.267	0.275	0.325
G2_SSR_	54	3.67	2.110	0.823	0.526	0.420
G3_SSR_	25	4.47	2.720	0.647	0.627	0.584
G4_SSR_	32	6.2	3.433	0.725	0.670	0.643
**Overall**	**158**	**7.27**	**2.331**	**0.616**	**0.524**	**0.544**
**SNP data**						
G1_SNP_	57	2	1.236	0.154	0.169	0.152
G2_SNP_	45	2	1.564	0.395	0.287	0.241
G3_SNP_	16	2	1.554	0.294	0.308	0.259
G4_SNP_	40	2	1.476	0.308	0.295	0.242
**Overall**	**158**	**2**	**1.427**	**0.288**	**0.264**	**0.230**

**Overall**: Refers to the genetic diversity parameters estimated for all samples using either SSR or SNP data, **N:** Number of individuals, **Na**: Number of alleles, **Ne**: Number of effective alleles, **H**_**obs**_: Observed Heterozygosity, **H**_**exp**_: Expected Heterozygosity, **PIC:** Polymorphic Information Content.

In spite of the small differences detected, group differentiation and genetic diversity parameters estimated with SSR data were mostly consistent with the ones calculated using SNP data. Providing more evidences for validating the new ddRADseq protocol exploited for cacao SNP genotyping.

## Discussion

### SSR and SNP genotyping

Cacao genetic studies, particularly cacao genotype classification, are key aspects for cacao agroindustry around the world. In the last decades, both SSR and SNP markers have been exploited for several applications in cacao. However, the search for new SNP genotyping method with improved potential as universal genotyping system remains and would constitute an important tool to face future challenges of cacao and chocolate industry [1,21,22]. In a previous work [24], we employed a new ddRADseq protocol for cacao plant genotyping to reveal the population structure and genetic diversity of cacao resources in eastern Cuba. This protocol differed from other GBS protocols used in cacao SNP genotyping [18,20,25] and it has been suggested the need to validate the SNP identified with GBS technologies such as ddRADseq, when high impact applications of the SNPs are expected [26,28]. Instead of validating individual SNPs, we compared the performances for population analysis of SNPs derived from this new SNP genotyping protocol with the dataset from a standard SSR genotyping method described for cacao and assayed in the past for studying Cuban traditional cacao plants [12,29]. These two methods differed in number of loci analyzed since only 15 international standard microsatellites were used for SSR fingerprinting while 7,880 high quality SNPs were identified in the 158 plants analyzed. Marker distribution along cacao genome also revealed some differences (S6 Table); remarkably no SSR marker belonged to chromosome 5; in contrast, 932 SNPs were identified in this location.

Few studies have comparatively assessed SSR and SNP markers in cacao; and most of them have been focused on clone classification and off-type identification [19,69–72]. Less attention has been paid to population structure analysis [22]. None of these works have used large SNP panels derived from a GBS protocol. For other crop species such as maize, it has been proposed to use from 7 to 11 times more SNPs than SSRs to reach similar results [67]. This value depends on the nature of the marker and the species itself. In our case, the SNP/SSR ratio was 525, raising questions about the ability of both datasets to produce similar results. Another constraint in our comparison was that, due to external restrictions, we basically employed different plants as cacao ancestry genetic group references for SSR and SNP marker analyses which also increased the uncertainty. Additionally, low percent of the 7,880 SNPs contained in the SNP dataset from ddRADseq experiments were found in other SNP panels with several application in cacao genetics [63,64], which suggested the variant combination found in our SNP dataset has not been previously explored for population analysis in cacao. However, the good quality of the datasets obtained with both marker systems constituted a proper starting point for the comparative performance of the genotyping systems as a way to validate the ddRADseq protocol exploited.

### Population analysis with SSR and SNP datasets

Model based clustering identified K = 2 and K = 4 as the most probable number of genetic groups, with STRUCTURE (SSR) and ADMIXTURE (SNPs) softwares, respectively. However, a good similarity was found among identified genetic groups from both SSR and SNP data based on cacao ancestries after assuming four genetic groups. This result was significantly important because, as mentioned previously, genotypes used as cacao ancestry genetic group references were different for both SSR and SNP panels. Additionally, their processing was also different: for SSR, references were genotyped as part of the experiments while for SNPs, DNA sequences data were downloaded and processed as described [16]. However, these reference datasets proved to be efficient in properly assigning reference plants in their expected cacao genetic group (S4 and S5 Tables, S1–S3 Figs).

STRUCTURE results depend among others on the number of populations sampled and the number of individuals in each population. Likewise, it accurately detects the uppermost hierarchical level of the population [46], leading in some cases to wrongly estimate the real number of genetic groups. In our case the plants were unevenly distributed among SSR groups: G1_SSR_ (47), G2_SSR_ (54), G3_SSR_ (25) and G4_SSR_ (32) which could affect STRUCTURE performance. Another important aspect was the presence of clonally-propagated individuals among the assayed ones, such as grafted plants of UF cacao clones. This could disturb the group identification process as it has been described that relatedness among samples influences the ability of STRUCTURE to correctly detect the genetic stratification especially when a low number of loci are used for the analysis [73,74]. Gutierrez et al. [22] compared SSRs and SNPs, to select SNP markers reflecting population origin for cacao and detected two genetic clusters among 420 plants from the 10 cacao ancestry genetic groups with a 219 SNP panel using Evanno method [46]; after re-analyzing the results with STRUCTURE SELECTOR software, nine genetic groups were identified, a result closer to what had been obtained with SSR data.

Both marker systems successfully detected Amelonado and Criollo ancestries as the more contributing ones with 66.8% and 74.7% of the total for SSR and SNP data, respectively. The prevalence of these two genetic groups in cacao cropping practices is known (76% of the samples came from cacao farms) and several studies using either SSRs or SNPs support these findings [9,14,75]. Other common ancestries detected with more than 5% average contribution in genetic groups using both marker systems were Contamana, Iquitos and Nanay while Nacional were detected only in SSR groups. The rest of the ancestries represented less than 5% of total group ancestry.

To further investigate in ancestry detection capabilities of both marker systems, we compared the ancestries of individual cacao clones: UF29, UF613, UF650, UF677 and SCA6, obtained from SSR and SNP genotyping as well as from publicly available data. A high correspondence was found between SNP-derived ancestries calculated by us and the data from either International Cacao Germplasm Database [49] or published ancestries of these clones from CATIE collection (Centro Agronómico Tropical de Investigación y Enseñanza) [17]. All these data were obtained using SNPs, though ICGD and CATIE employed significantly less SNPs (~ 40) than the 7,880 SNPs we exploited. The higher Nacional membership detected by our SNP dataset in UF29, UF613 and UF650 clones may be the result of differences in the cacao genome regions being surveyed by these SNPs for cacao ancestry membership estimation.

In most cases, cacao ancestry membership coefficients calculated from SSR data were slightly lower than those obtained from SNP data though they managed to identify the same type of ancestry among compared cacao clones and identified analogous genetic groups. The recognized disadvantages of each marker system may explain these results. On one hand, SSR genotyping system has a major drawback on allele size estimation because variations in size estimation between samples, runs, and equipment are possible [13]. The number of alleles identified in reference plants (127) was higher than among sampled plants (109), although more detailed analysis revealed a higher number of alleles in samples than in references for loci mTcCIR6 (11 vs 7) and mTcCIR8 (7 vs 6) as well as changes in size ranges between samples and references SSR data for loci: mTcCIR1, mTcCIR8, mTcCIR22, mTcCIR37 and mTcCIR4 (S5 Table). The combined effect of these factors may have led to the detection in all samples of a small contribution of the other cacao ancestries, apart from the more contributing ones. As a result, lower membership coefficients of the more contributing ancestries were obtained with SSRs in comparison with SNPs estimation.

On the other hand, reference plants data for SNP markers came from a whole genome sequencing study [16], different from the ddRADseq approach we used for SNP genotyping. This feature combined with the drawbacks associated to RADseq derived technologies, such as allele dropout, PCR duplicates and variations in coverage, may have led to errors in genotype and ancestry estimations [27,28]. The quality assessment performed, including the data supporting the identified SNPs, and the capacity of the SNPs to properly separate cacao reference plants into their expected cacao genetic groups supported a high quality of the SNP panel used; however, genotyping errors are still possible.

The disadvantages associated to SSR and SNP marker systems aforementioned along with the dissimilar marker distribution along cacao genome may explain the differences in cacao ancestry membership coefficients estimated from SSR and SNP data; but cacao ancestries identified from both data types still had an adequate level of correspondence. However, the provided evidence pointed out to a better performance of SNPs than SSRs for cacao ancestry estimation especially at individual level. Furthermore, these results support the claims for a universal cacao ancestry genetic group reference set as has been proposed elsewhere [21].

Identified genetic groups by model-based approaches were properly separated with principal coordinate analysis using genetic distances matrices based on allelic differences built from SSR and SNP data. A better grouping was obtained for SSR data especially for G1_SSR_ and G2_SSR_ groups in respect to G1_SNP_ and G2_SNP_ (Fig 3). The observed interference of some G1_SNP_ and G2_SNP_ plants into the other groups may be caused by a combination of factors: 1) the relax rule followed for group assignment allowed the inclusion of both high membership and admixed plants in the same genetic group, 2) the larger number of SNPs used for genotyping in comparison with SSR supports a higher genotype discriminant capacity of SNPs, putatively producing more disperse patterns and, last but not least, 3) the cacao farm planting policy in Cuba stimulates the use of seedlings from certified seeds, obtained by crossing specific cacao clones, such as UF650 (Amelonado/Criollo) x Pound-7 (Nanay) and UF677 (Amelonado/Criollo) x IMC-67 (Iquitos) [65]. These crossings would have led to the occurrence of hybrid progenies carrying highly mixed ancestry backgrounds in cacao farms like the one detected in G4 groups using both SSR and SNP data.

Principal coordinate analysis has been useful to confirm STRUCTURE clustering in other studies comparing SSRs and SNPs performance for population analysis in cacao and other crop species. In most cases, PCoA managed to properly separate the groups under study similarly to model-based clustering, though SNPs outperformed SSRs in some cases [67,68]. UPGMA dendrograms mostly agreed on already defined clusters, especially for G1 and G2 groups, though some subgroups were particularly evident within G3_SSR_, G4_SSR_ and G4_SNP_ samples. Plants forming these subgroups had some distinctive cacao ancestries pattern among them probably associated to the cacao farm planting policy previously discussed which could have favored their clustering together and apart from the other group members.

In general, the population analysis results obtained from SSR data were very consistent with the SNPs derived ones considering both the good congruence among the results of the different tools used to analyze the population with each marker system (Model-based approach, PCoA and UPGMA) and the optimal correspondence between the results from each tool using every marker system. Such level of consistency strongly supports a good performance of technologies exploited to generate the datasets used for the analysis and contributed to validate the ddRAseq protocol employed for the SNP genotyping of the cacao plants.

### Group differentiation and genetic diversity

AMOVA and F_ST_ pairwise comparison showed moderate to very large genetic differentiation among the identified genetic groups with both data types (F_ST_ SSR: 0.072–0.283 and F_ST_ SNP: 0.087–0260). Lower differentiation was reported by Gutierrez et al. [22] when comparing SSR and SNP markers in cacao: F_ST_ values, based on a 219 SNP panel, ranged from 0.038 to 0.194 even though only cacao ancestry genetic group reference plants were used. This result differs to what we achieved when assessing the differentiation of cacao ancestry genetic group references for both data types (S4 and S5 Tables). Genetic diversity based on SSRs and SNPs were estimated by calculating H_obs_, H_exp_ and PIC. SSR diversity parameters were higher than with SNP markers; such behavior has been described in other studies with goals similar to this one [66–68]. The differences in genetic diversity estimation could be explained by the higher number of alleles, frequently detected in SSR markers compared to the bi-allelic nature of SNPs used in most population studies. It has been suggested that mechanisms supporting SNP diversity (point mutations) are slower than the replication slippage behind SSR diversity justifying the higher number of alleles per loci in SSRs [13].

Per groups genetic diversity parameters showed the lowest genetic diversity, as revealed by Ne, H_obs_, H_exp_ and PIC values, in groups containing the highest contribution from Amelonado ancestry (G1_SSR_ and G1_SNP_). Amelonado genetic group plants are characterized by being highly homozygous and self-compatible, hence low genetic diversity should be expected among plants of this group [34]. On the contrary, highest H_obs_ values came from G2_SSR_ and G2_SNP_. These groups were mainly formed by Amelonado and Criollo hybrids. This type of cacao plants is usually recognized as Trinitario type and is widely recognized they surged from the crossing between Amelonado and Criollo plants. Criollo plants are also highly homozygous; therefore, the high H_obs_ values found in G2_SSR_ and G2_SNP_ are congruent with the heterozygous nature expected for Trinitario plants [5,7,76].

The comparison of SSR and SNP markers for genetic differentiation and genetic diversity assessment of the 158 genotyped plants also showed very consistent results to each other, as with the other population analysis tools. These results confirmed the good performances of both systems for cacao genotyping and validated the ddRADseq protocol used for that purpose. The use of GBS protocols for cacao classification has additional advantages, as with other crop species, since other applications are possible such as association studies, genomic selection, gene and QTL mapping. So far, the strategy followed for the identification of a SNP panel useful in cacao genetic classification has been focused on selecting a small number of highly polymorphic SNPs able to properly separate references of cacao ancestry genetic groups [22]. The continuous advances in sequencing technology could provide the mean for a paradigm change in which few effective GBS protocols, like the one here described, may be available for cacao genetic studies and could be selected depending on research goals. Whatever the case, more efforts are required to provide the cacao research community with universal reliable SNP genotyping tools to face current and future challenges of cacao agroindustry.

## Conclusions

The high consistency among the results of the population analysis in *Theobroma cacao* using the SNP dataset derived from ddRADseq experiments and the genotypic data from the 15 international standard SSR markers validated the ddRADseq protocol for cacao SNP genotyping. The low coincidence between the variant sites of the SNP dataset here obtained and other SNP panels reported for cacao supports the variant sites contained in our SNP dataset have been poorly exploited for cacao genetic studies. Therefore, these SNPs and the ddRADseq protocol could constitute an opportunity to explore new approaches for the development of a new SNP-based genotyping system for cacao classification as well as for other applications in cacao genetic studies.

## Supporting information

S1 Appendix SSR genotypic dataset. (XLSX)

S1 Table Details of the cacao farms used for sampling purpose. (PDF)

S2 Table List of clones used as references of cacao ancestry genetic groups for SSR data. (PDF)

S3 Table List of clones used as references of cacao ancestry genetic groups for SNPs data, adjusted from Cornejo et al. [16].
(PDF)

S4 Table AMOVA results of cacao references of ancestry genetic groups using SSR and SNPs data. (PDF)

S5 Table F_ST_ pairwise comparison among references of cacao ancestry genetic groups according to Motamayor et al. [6] using their genotypes from SSR and SNP data.
(PDF)

S6 Table SSR loci description among samples and references of cacao ancestry genetic groups. (PDF)

S7 Table Transition and transversion statistics of SNPs data vs published. (PDF)

S8 Table SSR and SNPs counting on per chromosome. (PDF)

S1 Fig Membership to cacao ancestry genetic groups of reference plants using SSR and SNPs data. (PDF)

S2 Fig Dendrograms of cacao ancestry genetic group references using SSR and SNP markers. (PDF)

S3 Fig Principal coordinate analysis bi-plots of cacao ancestry genetic group references. (PDF)

S4 Fig Per SNP distribution of hard filtering variables. (PDF)

S5 Fig Heatmap of SNPs density per chromosome. (PDF)
