# Supplementary material for: Comparing the performances of SSR and SNP markers for population analysis in Theobroma cacao L., as alternative approach to validate a new ddRADseq protocol for cacao genotyping
Source: PLoS One. 2024 May 31;19(5):e0304753. doi: 10.1371/journal.pone.0304753 (PMC11142705; doi:10.1371/journal.pone.0304753)
Supplement: S1 Table — (PDF) [file pone.0304753.s002.pdf]

Supporting Table 1. Details of the cacao farms used for sampling purpose.

Farm ID	Productive Pole	Farm Name	Cacao plants origin	North Coordinate	West Coordinate	Farm Size (ha)	Plot (Unit)	Sampled Plant (Unit)
F08	Jamal	Finca Los Yaser	Traditional / Hybrid / Grafted	20°16,413' N	74°25,644' W	16	8	40
F15	Jamal	Santa Rita	Traditional / Hybrid / Grafted	20°16,638' N	74°25,521' W	9,45	5	25
F02	San Luis	Finca Santa María	Traditional / Hybrid	20°18,73' N	74°25,61' W	11,7	3	15
F19	San Luis	La Esperanza I	Grafted	20°17,698' N	74°26,779' W	3	3	15
F05	Paso de Cuba / Sabanilla	Finca Elcita	Hybrid / Grafted	20°17,101' N	74°27,907' W	5,33	3	15
F10	Paso de Cuba / Sabanilla	Finca San Miguel	Hybrid / Grafted	20°15,577' N	74°27,713' W	6,66	3	15
F11	Paso de Cuba / Sabanilla	Poca Pena	Grafted	20°15,192' N	74°27,625' W	8,5	4	20

Note: Cacao plants origin: Cacao plant type based on the reproduction mode according to farmers. **Farm size** refers to total amount of hectare of the farm not only to the cacao planted area. **Plot** is number of plots raised in the cacao plantation. **Sampled Plant** refers to the number of plants collected for analysis purposes in the farm**.**
