# Supplementary material for: Comparing the performances of SSR and SNP markers for population analysis in Theobroma cacao L., as alternative approach to validate a new ddRADseq protocol for cacao genotyping
Source: PLoS One. 2024 May 31;19(5):e0304753. doi: 10.1371/journal.pone.0304753 (PMC11142705; doi:10.1371/journal.pone.0304753)
Supplement: S2 Table — (PDF) [file pone.0304753.s003.pdf]

Supporting Table 2. List of clones used as references of cacao ancestry genetic groups for SSR data.

ID	Plant Code	Source	Genetic group
C06	Amelonado 9(11)	CATIE	Amelonado
C08	Amelonado 13(14)	CATIE	
C39	SIAL 407	CIRAD	
C13	SCA 12	INAF/Cuba	Contamana
C30	SCA 9	ICGD	
C38	SCA 11	ICGD	
C01	Criollo 23	CATIE	Criollo
C15	CRIOLLO 22 [CRI]	ICGD	
C17	LCTEEN 163/A	ICGD	Curaray
C18	LCTEEN 241	ICGD	
C09	ELP 20/A	CATIE	Guiana
C10	ELP 16/A	CATIE	
C11	B7 B3	CATIE	
C12	B7 A2	CATIE	
C32	GU 285A	CIRAD	
C34	KER 3	CIRAD	
C14	AMAZ 12 [CHA]	ICGD	Iquitos
C16	IMC 103	ICGD	
C27	POUND 12/A [POU]	ICGD	
C33	IMC 68	CIRAD	
C21	PA 120 [PER]	ICGD	Marañón
C22	PA 121 [PER]	ICGD	
C23	PA 136 [PER]	ICGD	
C24	PA 169 [PER]	ICGD	
C25	PA 4 [PER]	ICGD	
C36	PA 120	CIRAD	
C37	PA 121	CIRAD	
C19	NA 149	ICGD	Nanay
C20	NA 232	ICGD	
C26	POUND 10/B [POU]	ICGD	
C35	NA 127	CIRAD	
C07	LCTEEN 46	CATIE	Nacional
C31	U 26 [PER]	ICGD	
C28	RB 46 [BRA]	ICGD	Púrus
C29	RB 47 [BRA]	ICGD	

ID: Identifier used in this study, **Plant Code:** Code according to the plant supplier, **Source:** Supplier of the leaves of cacao reference clones: **CATIE:** Centro Agronómico Tropical de Investigación y Enseñanza (Costa Rica), **ICGD:** International Cocoa Germplasm Database (UK) and INAF: Instituto de Investigaciones Agroforestales (Cuba). **Genetic Group:** Plant memberships to cacao ancestry genetic groups defined by Motamayor et al. [6]. These plants were processed the same way as the 158 samples to get SSR profile from the 15 international standard microsatellites for cacao DNA fingerprint.
