# Supplementary material for: Comparing the performances of SSR and SNP markers for population analysis in Theobroma cacao L., as alternative approach to validate a new ddRADseq protocol for cacao genotyping
Source: PLoS One. 2024 May 31;19(5):e0304753. doi: 10.1371/journal.pone.0304753 (PMC11142705; doi:10.1371/journal.pone.0304753)
Supplement: S3 Table — [16]. (PDF) [file pone.0304753.s004.pdf]

Supporting Table 3. List of clones used as references of cacao ancestry genetic groups for SNPs data, adjusted from Cornejo et al. [16].

ID	Plant Code	Genetic Group
crio01	Criollo	Criollo
crio02	Sp1	
crio03	Sp3	
crio04	Sp9	
cura05	Cur 3 G39 - A10	Curaray
cura06	Cur 3 G37 - A6	
cura07	Cur 3 G38 - A8	
cura08	LCTEEN - 141	
cura09	SIL -1-G56-A6	
nana10	SPEC - 194 75	Nanay
nana11	Pound 7	
nana14	Pound 7B	
nana15	Pound 10-B	
nana16	NA – 92	
nana17	NA – 331	
nana18	NA – 286	
nana19	NA – 702	
cont20	NH – 53	Contamana
cont21	NH – 40	
cont22	T 695 - SCA6 - A1	
cont24	SCA 24.2	
cont26	SCA -11	
cont27	PMF – 27	
cont28	PMF – 20	
amel29	TRD86	Amelonado
amel31	REDAMEL 1-31	
amel32	SIAL 84	
amel33	SIAL 70	
amel34	SIC 806	
amel35	mvP30	
amel36	SIAL 169	
amel37	Matina	
amel38	Matina Tica 2	
amel39	Catongo	
mara40	PA – 218	Marañón
mara42	PA – 150	
mara43	PA – 51	
mara44	PA – 107	
mara45	PA – 169	
mara46	MO – 4	
mara47	MO – 9	
mara48	PA 289	
mara50	PA -56	
mara52	PA -121	
naci54	UF 273 T1	Nacional
naci55	UF 273 T2	
naci56	AM -1 -54	
naci57	Brisas -1	
guia58	GU - 308[a]	Guiana
guia60	GU -300 P	
guia61	GU – 222	
guia62	GU - 291F	
guia63	GU - 175P	
guia64	GU - 114P	
guia65	GU - 255V	
iqui67	IMC – 51	Iquitos
iqui69	IMC – 12	
iqui70	IMC – 50	
iqui71	IMC – 20	
iqui72	IMC -67	
iqui73	IMC – 14	
puru75	CAB 77 - PL5	Purús
puru76	CAB 76 - PL3	
puru77	RB 47 - PL3	
puru79	RB 39 - PL1	

ID: Identifier used in this study, **Plant Code:** Code as Cornejo *et al.* (2018), **Genetic Group:** Plant memberships to cacao ancestry genetic groups defined by Motamayor et al. [6] and assigned by Cornejo et al. [16]. Sequence data of these plants were downloaded from NCBI (*BioProject* PRJNA486011) using *SRA toolkit* v2.11.0 (SRA Toolkit Development Team, 2022) and processed according to [16] to obtain the Reference SNP dataset.
