# Supplementary material for: Comparing the performances of SSR and SNP markers for population analysis in Theobroma cacao L., as alternative approach to validate a new ddRADseq protocol for cacao genotyping
Source: PLoS One. 2024 May 31;19(5):e0304753. doi: 10.1371/journal.pone.0304753 (PMC11142705; doi:10.1371/journal.pone.0304753)
Supplement: S4 Table — (PDF) [file pone.0304753.s005.pdf]

Supporting Table 4. AMOVA results of cacao references of ancestry genetic groups using SSR and SNPs data.

Source of Variation	SSR					SNPs				
	Df	SS	MS	Sigma	Variation (%)	Df	SS	MS	Sigma	Variation (%)
Between Groups	9	217.56	24.17	6.36	71.99	9	85,295.20	9,477.24	1,410.59	76.83
Within Groups	25	61.89	2.48	2.48	28.01	55	23,395.75	425.38	425.38	23.17
Total	34	279.45	8.22	8.84	100.00	64	108,690.96	1,698.30	1,835.96	100.00

Df: Degree of freedom, **SS:** Square Sum, **MS:** Mean Square. Highly significant values with $p < 0.001$.
