# Supplementary material for: Comparing the performances of SSR and SNP markers for population analysis in Theobroma cacao L., as alternative approach to validate a new ddRADseq protocol for cacao genotyping
Source: PLoS One. 2024 May 31;19(5):e0304753. doi: 10.1371/journal.pone.0304753 (PMC11142705; doi:10.1371/journal.pone.0304753)
Supplement: S5 Table — [6] using their genotypes from SSR and SNP data. (PDF) [file pone.0304753.s006.pdf]

Supporting Table 5. F_{ST} pairwise comparison among references of cacao ancestry genetic groups according to Motamayor et al. [6] using their genotypes from SSR and SNP data.

SSR

	Amel	Cont	Crio	Cura	Guia	Iqui	Mara	Nana	Naci
Cont	0.865								
Crio	0.869	0.779							
Cura	0.725	0.617	0.550						
Guia	0.789	0.769	0.733	0.669					
Iqui	0.502	0.473	0.458	0.275	0.516				
Mara	0.460	0.593	0.615	0.485	0.527	0.240			
Nana	0.699	0.722	0.733	0.528	0.731	0.349	0.416		
Naci	0.648	0.514	0.528	0.265	0.654	0.239	0.485	0.557	
Puru	0.780	0.721	0.680	0.440	0.680	0.387	0.514	0.633	0.401

SNPs

	Amel	Cont	Crio	Cura	Guia	Iqui	Mara	Nana	Naci
Cont	0.710								
Crio	0.925	0.765							
Cura	0.803	0.467	0.835						
Guia	0.750	0.619	0.924	0.749					
Iqui	0.570	0.451	0.776	0.516	0.558				
Mara	0.478	0.495	0.791	0.570	0.392	0.381			
Nana	0.621	0.630	0.878	0.717	0.683	0.374	0.480		
Naci	0.703	0.395	0.799	0.454	0.656	0.398	0.467	0.626	
Puru	0.724	0.452	0.849	0.584	0.654	0.392	0.424	0.587	0.487

Amel: Amelonado, **Cont:** Contamana, **Crio:** Ciollo, **Cura:** Curaray, **Guia:** Guiana, **Iqui:** Iquitos, **Mara:** Marañón, **Nana:** Nanay, **Naci:** Nacional and **Puru:** Purús. All F_{ST} values were significant ($p < 0.00$
