# Supplementary material for: Comparing the performances of SSR and SNP markers for population analysis in Theobroma cacao L., as alternative approach to validate a new ddRADseq protocol for cacao genotyping
Source: PLoS One. 2024 May 31;19(5):e0304753. doi: 10.1371/journal.pone.0304753 (PMC11142705; doi:10.1371/journal.pone.0304753)
Supplement: S6 Table — (PDF) [file pone.0304753.s007.pdf]

Supporting Table 6. SSR loci description among samples and references of cacao ancestry genetic groups.

Locus	Samples			References		
	Na	Range	Miss (%)	Na	Range	Miss (%)
mTcCIR1	5	119-141	0	6	129-147	0
mTcCIR6	11	225-251	0	7	221-249	0
mTcCIR7	4	154-160	0	5	154-162	0
mTcCIR8	7	280-306	0	6	290-306	0
mTcCIR11	6	290-316	2.53	12	290-316	5.7
mTcCIR12	7	188-222	0	9	188-253	0
mTcCIR15	8	234-258	0	10	234-260	0
mTcCIR18	7	332-356	0	8	332-348	0
mTcCIR22	5	275-292	0	6	281-299	0
mTcCIR24	6	186-204	0	7	174-204	0
mTcCIR26	5	285-305	0	7	285-309	0
mTcCIR33	11	275-347	0	11	275-347	0
mTcCIR37	9	134-186	0	12	134-178	0
mTcCIR40	10	248-286	0	12	258-288	2.9
mTcCIR60	8	188-216	0	9	188-216	0
**Total**	**109**	**-----**	**0.17**	**127**	**-----**	**0.57**

Na: number of alleles per locus, **Range:** alleles size range per locus, **Miss:** missingness on per locus basis.
