# Supplementary material for: Comparing the performances of SSR and SNP markers for population analysis in Theobroma cacao L., as alternative approach to validate a new ddRADseq protocol for cacao genotyping
Source: PLoS One. 2024 May 31;19(5):e0304753. doi: 10.1371/journal.pone.0304753 (PMC11142705; doi:10.1371/journal.pone.0304753)
Supplement: S7 Table — (PDF) [file pone.0304753.s008.pdf]

Supporting Table 7. Transition and transversion statistics in SNPs data vs published.

**Change (Type)**	**SNPs data**		**Published**	
	**Count**	**%**	**Count**	**%**
Total SNPs	7,880	100	12,357	100
C/T	2,467	31.3	3,894	31.5
A/G	2,488	31.6	3,856	31.2
**Ts**	**4655**		**7,750**	
C/G	543	6.9	758	6.1
G/T	770	9.8	1,203	9.7
A/C	749	9.5	1,243	10.1
A/T	863	10.9	1,403	11.4
**Tv**	**2925**		**4,607**	
**Ts/Tv**	**1.591**		**1.682**	

Change: Refers to the substitution type, **Count:** numbers of changes, **C:** Cytosine, **T:** Thymine, **A:** Adenine, **G:** Guanine, **Ts:** Transitions, **Tv:** Transversions, **Ts/Tv:** Transitions/Transversions ratio. **Published** refers to data published by Osorio-Guarin et al. [62].
