# Supplementary material for: Comparing the performances of SSR and SNP markers for population analysis in Theobroma cacao L., as alternative approach to validate a new ddRADseq protocol for cacao genotyping
Source: PLoS One. 2024 May 31;19(5):e0304753. doi: 10.1371/journal.pone.0304753 (PMC11142705; doi:10.1371/journal.pone.0304753)
Supplement: S8 Table — (PDF) [file pone.0304753.s009.pdf]

Supporting Table 8. SSR and SNPs counting on per chromosome.

Chr	SSR count	SNP count
1	2	1,044
2	2	1,080
3	1	803
4	3	804
5	0	932
6	1	630
7	1	506
8	2	534
9	2	985
10	1	562
**Total**	**15**	**7,880**

Chr: Cacao chromosome number, **SSR count:** number of SSR, **SNP count:** number of SNP
