# Supplementary material for: Comparing the performances of SSR and SNP markers for population analysis in Theobroma cacao L., as alternative approach to validate a new ddRADseq protocol for cacao genotyping
Source: PLoS One. 2024 May 31;19(5):e0304753. doi: 10.1371/journal.pone.0304753 (PMC11142705; doi:10.1371/journal.pone.0304753)
Supplement: S1 Fig — (PDF) [file pone.0304753.s010.pdf]

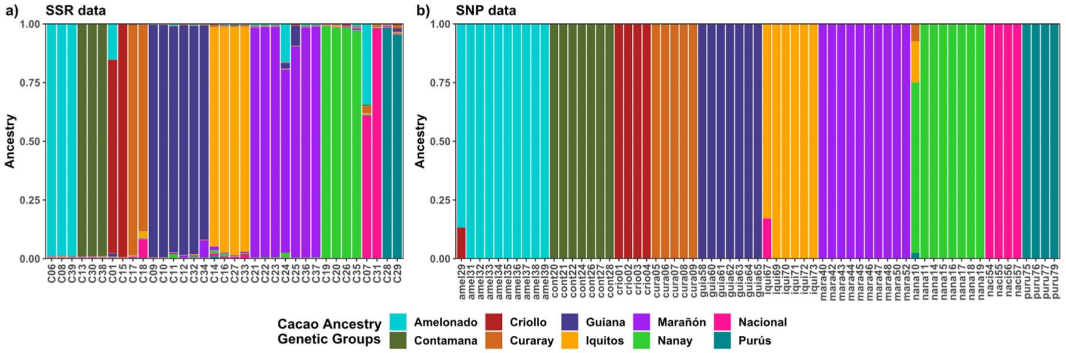

Supporting Figure 1. Membership to cacao genetic groups of reference plants using SSR (a) and SNPs (b) data. STRUCTURE and ADMIXTURE softwares were used for group identification for SSR and SNP markers, respectively. Plot were generated using ggplot2 package from R program.
