# Supplementary material for: Comparing the performances of SSR and SNP markers for population analysis in Theobroma cacao L., as alternative approach to validate a new ddRADseq protocol for cacao genotyping
Source: PLoS One. 2024 May 31;19(5):e0304753. doi: 10.1371/journal.pone.0304753 (PMC11142705; doi:10.1371/journal.pone.0304753)
Supplement: S2 Fig — (PDF) [file pone.0304753.s011.pdf]

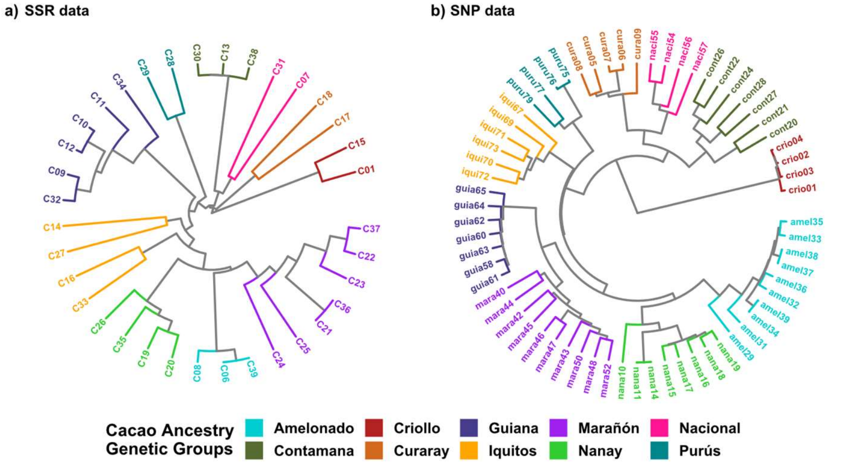

Supporting Figure 2. Dendrograms of cacao ancestry genetic group references using SSR (a) and SNP (b) markers. Clustering by UPGMA method from genetic distance matrices based on plant allelic differences. Plot were generated using ggtree and treeio packages from R program.
