# Supplementary material for: Comparing the performances of SSR and SNP markers for population analysis in Theobroma cacao L., as alternative approach to validate a new ddRADseq protocol for cacao genotyping
Source: PLoS One. 2024 May 31;19(5):e0304753. doi: 10.1371/journal.pone.0304753 (PMC11142705; doi:10.1371/journal.pone.0304753)
Supplement: S3 Fig — (PDF) [file pone.0304753.s012.pdf]

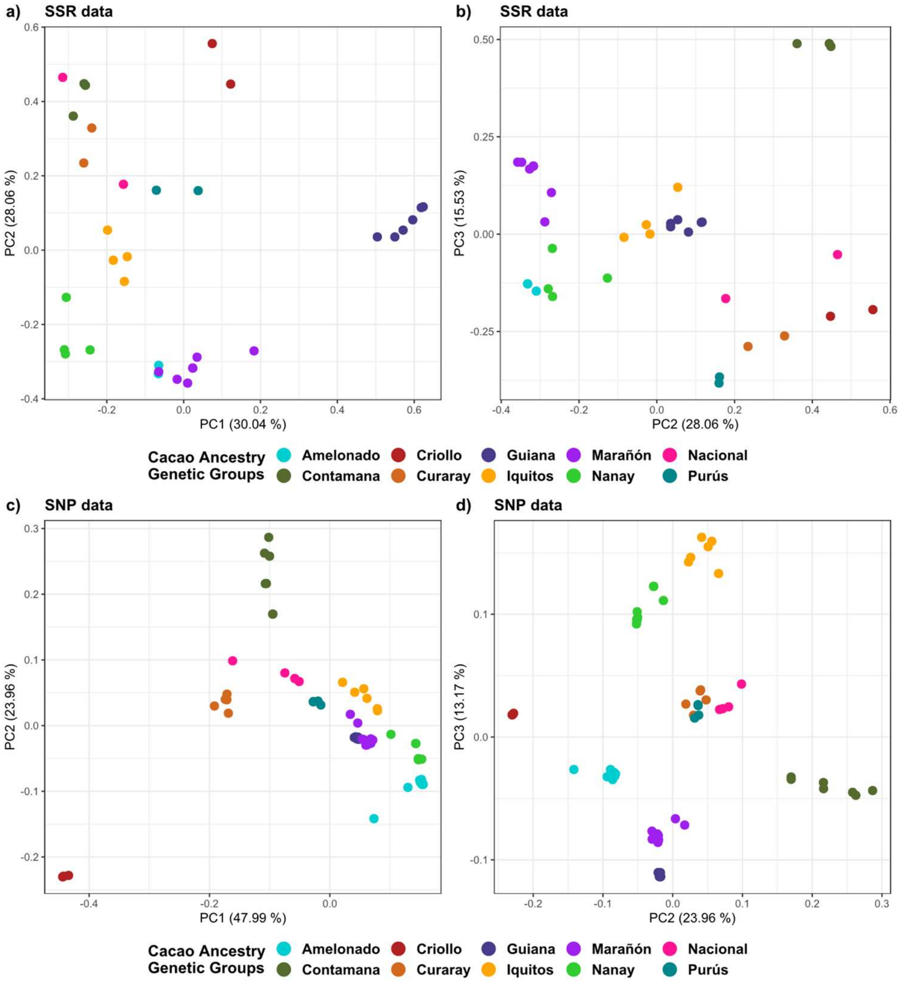

Supporting Figure 3. Principal coordinate analysis bi-plots of cacao ancestry genetic group references. Using SSR (3a and 3b) and SNP data (3c and 3d). Plots were generated using ggplot2 and ggpubr packages from R program.
