# Supplementary material for: Comparing the performances of SSR and SNP markers for population analysis in Theobroma cacao L., as alternative approach to validate a new ddRADseq protocol for cacao genotyping
Source: PLoS One. 2024 May 31;19(5):e0304753. doi: 10.1371/journal.pone.0304753 (PMC11142705; doi:10.1371/journal.pone.0304753)
Supplement: S4 Fig — (PDF) [file pone.0304753.s013.pdf]

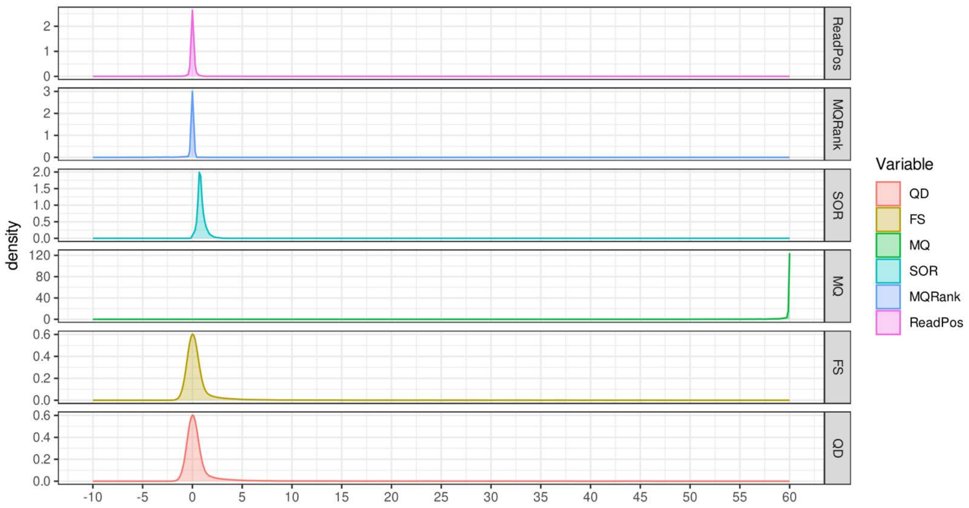

Supporting Figure 4. Per SNP distribution of hard filtering variables. **QD** (*Quality by Depth*), **MQ** (*Mapping Quality*), **FS** (*Fisher Strand*), **SOR** (S*trand Odds Ratio*), **MQRank** (*Mapping Quality Rank Sum Test*) and **ReadPos** (*Read Position Rank Sum Test*) from the final SNP dataset. Plots were built using ggplot R package. **QD** values should be higher than 2 with peaks around 12 and 32. **FS**, **MQRank**, y **ReadPos** quantified the bias in SNPs identification, values closed to 0 are desired. **MQ** is the Mapping Quality (MAPQ) average of the reads supporting the variant sites and the values around 60 (the maximum) are expected. **SOR** values should be between 0 and 3. References values were taken from: "Hard-filtering germline short variants" at GATK Technical Documentation [39].
