# Supplementary material for: Comparing the performances of SSR and SNP markers for population analysis in Theobroma cacao L., as alternative approach to validate a new ddRADseq protocol for cacao genotyping
Source: PLoS One. 2024 May 31;19(5):e0304753. doi: 10.1371/journal.pone.0304753 (PMC11142705; doi:10.1371/journal.pone.0304753)
Supplement: S5 Fig — (PDF) [file pone.0304753.s014.pdf]

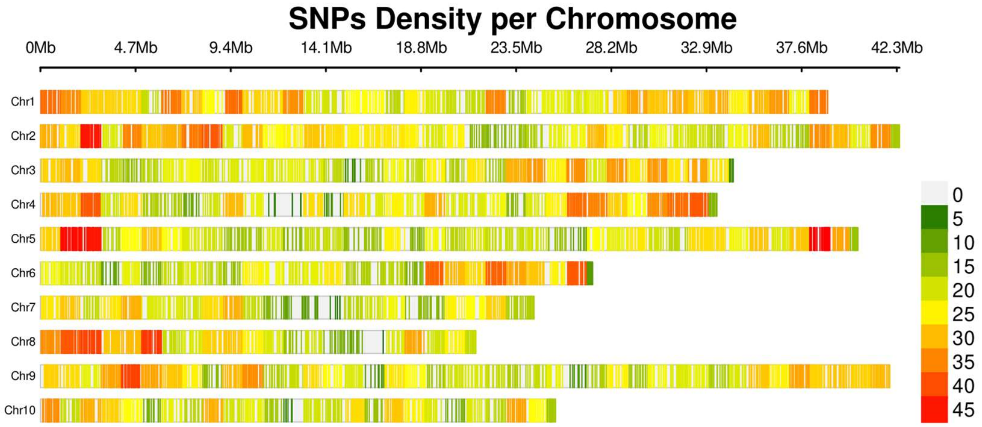


**Supporting Figure 5.** Heatmap of SNPs density per chromosome. A window size of 1 Mb was set for counting and plotting purposes. The plots were generated using the function CMPlot of the R package with the same name.
